# Identification of natural antimicrobial peptides from bacteria through metagenomic and metatranscriptomic analysis of high-throughput transcriptome data of Taiwanese oolong teas

**DOI:** 10.1186/s12918-017-0503-4

**Published:** 2017-12-21

**Authors:** Kai-Yao Huang, Tzu-Hao Chang, Jhih-Hua Jhong, Yu-Hsiang Chi, Wen-Chi Li, Chien-Lung Chan, K. Robert Lai, Tzong-Yi Lee

**Affiliations:** 1 Department of Computer Science and Engineering, Yuan Ze University, Taoyuan City, 320 Taiwan; 2 Department of Medical Research, Hsinchu Mackay Memorial Hospital, Hsinchu City, 300 Taiwan; 3 Graduate Institute of Biomedical Informatics, Taipei Medical University, Taipei, 110 Taiwan; 4 Department of Information Management, Yuan Ze University, Taoyuan City, 320 Taiwan; 5 Innovation Center for Big Data and Digital Convergence, Yuan Ze University, Taoyuan, city, 320 Taiwan

**Keywords:** Antimicrobial peptide, Amp, Next-generation sequencing, Metagenomics, Metatranscriptomics, Oolong teas

## Abstract

**Background:**

Anti-microbial peptides (AMPs), naturally encoded by genes and generally containing 12–100 amino acids, are crucial components of the innate immune system and can protect the host from various pathogenic bacteria and viruses. In recent years, the widespread use of antibiotics has resulted in the rapid growth of antibiotic-resistant microorganisms that often induce critical infection and pathogenesis. Recently, the advent of high-throughput technologies has led molecular biology into a data surge in both the amount and scope of data. For instance, next-generation sequencing technology has been applied to generate large-scale sequencing reads from foods, water, soil, air, and specimens to identify microbiota and their functions based on metagenomics and metatranscriptomics, respectively. In addition, oolong tea is partially fermented and is the most widely produced tea in Taiwan. Many studies have shown the benefits of oolong tea in inhibiting obesity, reducing dental plaque deposition, antagonizing allergic immune responses, and alleviating the effects of aging. However, the microbes and their functions present in oolong tea remain unknown.

**Results:**

To understand the relationship between Taiwanese oolong teas and bacterial communities, we designed a novel bioinformatics scheme to identify AMPs and their functional types based on metagenomics and metatranscriptomic analysis of high-throughput transcriptome data. Four types of oolong teas (Dayuling tea, Alishan tea, Jinxuan tea, and Oriental Beauty tea) were subjected to 16S ribosomal DNA and total RNA extraction and sequencing. Metagenomics analysis results revealed that Oriental Beauty tea exhibited greater bacterial diversity than other teas. The most common bacterial families across all tea types were *Bacteroidaceae* (21.7%), *Veillonellaceae* (22%), and *Fusobacteriaceae* (12.3%). Metatranscriptomics analysis results revealed that the dominant bacteria species across all tea types were *Escherichia coli*, *Bacillus subtilis*, and *Chryseobacterium sp. StRB126*, which were subjected to further functional analysis. A total of 8194 (6.5%), 26,220 (6.1%), 5703 (5.8%), and 106,183 (7.8%) reads could be mapped to AMPs.

**Conclusion:**

We found that the distribution of anti-gram-positive and anti-gram-negative AMPs is highly correlated with the distribution of gram-positive and gram-negative bacteria in Taiwanese oolong tea samples.

**Electronic supplementary material:**

The online version of this article (10.1186/s12918-017-0503-4) contains supplementary material, which is available to authorized users.

## Background

Anti-microbial peptides (AMPs), naturally encoded by genes and generally consisting of 12–100 amino acids, are crucial components of the innate immune system and can protect the host from various pathogenic bacteria and viruses [1]. In recent years, the widespread use of antibiotics has resulted in the rapid growth of antibiotic-resistant microorganisms that often induce critical infection and pathogenesis. Because of their broad-spectrum antimicrobial activities, AMPs are active against a variety of pathogens, such as gram-positive and gram-negative bacterial, fungi, viruses, and parasites [2]. Thus, it is important to identify natural AMPs for the development of new antibiotics. Many approaches have been proposed for the development of potential drugs, such as in silico prediction of AMPs based on protein sequences. Currently, more than 3900 natural AMPs have been identified in plants and animals [3]. In a previous study, Wan et al. [4] found that green tea possessed high antimicrobial activity against *Escherichia coli* by inducing the secretion of plant antimicrobial peptides.

Teas can be classified according to their degree of fermentation: non-fermented green tea, partially fermented oolong tea, completely fermented black tea, and post-fermented dark tea [5]. Oolong tea is the highest yielding tea in Taiwan, accounting for over 90% of total tea production annually. Previous studies have reported that oolong tea can inhibit obesity [6], reduce dental plaque deposition [7], antagonize allergies [8], and moderate aging [9]. Investigations of microbes in Puer tea have been reported previously by Wen et al. [10], Zhou et al. [11], and Xu et al. [12], who showed that *Candida* and *Aspergillus niger* were the dominant microbes in Puer tea. However, the microbes present in oolong teas have not been identified and it is unknown which AMPs are produced by bacteria in oolong tea.

Recently, the advent of high-throughput technologies has led molecular biology into a data surge in both the growth and scope of data. For instance, next-generation sequencing (NGS) technology has been applied to generate large-scale sequencing reads from foods, water, soil, air, and specimens to identify microbiota and their functions based on metagenomics and metatranscriptomics, respectively. Additionally, mass spectrometry is widely applied in proteomics studies to detect thousands of peptides in one experiment.

The emergence of NGS technology has enabled analysis of genetic materials obtained directly from the environment and examination of biological diversity in a sensitive and efficient manner that it not possible using traditional approaches. While metagenomics studies target species diversity at the DNA level, metatranscriptomics analyses are used to investigate the activities and interactions among microbial communities in the extracted environment based on expression profiles [13]. Metagenomics and metatranscriptomics analyses of diverse microscopic organisms in their natural environments, including the human body, have revolutionized the understanding of the relationships between microbes and their hosts. Compared with functional gene microarrays, metatranscriptomic sequencing can detect gene transcripts without the restriction of targeting a specific species in complicated biological systems. Furthermore, without the noise associated with hybridization signals, discrete output of metatranscriptomic sequencing enables analysis of fine-scale variations in transcript sequences [14]. Metatranscriptomic sequencing has been applied to different levels. For example, Jung et al. [15] profiled the metatranscriptome of microbial species active during kimchi fermentation. Marchetti et al. [16] and Mason et al. [17] sequenced the transcriptomes of ocean microbes to identify active members their functional responses after environmental changes. Maurice et al. [18] conducted metatranscriptome profiling, 16S rRNA gene sequencing, and flow cytometry to identify dominant bacterial species in the human gut microbiota as well as the physiology and gene expression responses of bacteria to xenobiotics. John et al. [14] showed that Illumina sequencing could detect more significant differential genes than microarray; after qPCR validation, the difference in gene expression from sequencing data was found to be more consistent with those of real biological situations. Thus, RNA-seq analysis is less restricted than microarray and provides more gene expression information.

The relationship between microbial species and humans has been reported previously. For example, Arumugam et al. [19] revealed that Firmicutes and Bacteroidetes were major groups of human intestinal microbiota, Ley et al. [20] showed that Firmicutes and Bacteroidetes were human gut microbes associated with obesity, Kostic et al. [21] found that the number of Fusobacteria in colon cancer cells was higher than in healthy colon tissues, and Scheperjans et al. [22] showed that the number of bacteria from *Prevotellaceae* in patients with Parkinson’s disease was much lower than in the normal gut. In contrast, the microbes present in oolong tea and their functions remain unknown.

Rapidly advancing technologies have enabled examination of the genome, transcriptome, and proteome in a comprehensive manner. However, extracting meaningful information from large amounts of data and evaluating biological functions from a systems biology perspective are very challenging in bioinformatics studies. Therefore, to understand the distribution of microbiota and their potential functions in oolong teas, we conducted metagenomic and metatranscriptomic sequencing of four different Taiwanese oolong teas: Dayuling tea, Alishan tea, Jinxuan tea, and Oriental Beauty tea. Dayuling tea, Alishan tea, Jinxuan tea, and Oriental Beauty tea differ in their regions of origin and production processes. Dayuling tea, Alishan tea, and Jinxuan tea are lightly fermented high-mountain teas produced with varying degrees of roasting: non-roasted, medium roast, and light roast, respectively. In contrast, Oriental Beauty tea is a heavily fermented, light-roast tea and is made from tea leaves infested with *Jacobiasca formosana* [23]; thus, this tea may contain commensal microbial communities that differ from those in Dayuling tea, Alishan tea, and Jinxuan tea.

The aims of this study were to identify the dominant microbial species and their potential functions and identify AMPs and their functional types in different oolong teas. We developed a novel bioinformatics method for identifying AMPs and their functional types based on metagenomics and metatranscriptomic analysis of high-throughput transcriptome data. This is the first study to analyze microbial diversity in Taiwanese oolong teas using metagenomic and metatranscriptomic approaches.

## Methods

### DNA and RNA extraction

Three grams of green tea leaves were mixed with 150 mL of tap water and DNA and RNA were extracted from the mixture. The QIAamp DNA Blood Mini Kit (Qiagen, Hilden, Germany) was used for DNA extraction. Each sample was transferred to a 1.5-mL microcentrifuge tube and centrifuged at 13,000 rpm for 2 min to pellet the bacteria. Bacterial pellets were suspended in 180 mL of an appropriate enzyme solution and incubated for at least 30 min at 37 °C. Next, 20 mL proteinase K and 200 mL Buffer AL were added to the sample and mixed by vortexing. Each suspension was incubated at 56 °C for 30 min and then for an additional 15 min at 95 °C. The sample was briefly centrifuged to pellet the suspension. After this, extraction was conducted following the protocol of the QIAamp DNA Blood Mini Kit. DNA was eluted with 30 mL Buffer AE and centrifuged at 8000 rpm for 1 min. The DNA extract was stored at 220 °C until further analysis.

For RNA extraction, 0.5 mL of 100% isopropanol was added to the aqueous phase and then incubated at room temperature for 10 min. This sample was centrifuged at 12,000×*g* for 10 min at 4 °C and the supernatant was removed from the tube, leaving only the RNA pellet. The RNA pellet was washed with 1 mL of 75% ethanol and then vortexed to mix. Following centrifugation at 7500×*g* for 5 min at 4 °C, the supernatant was discarded and the RNA pellet was air-dried for 10 min. The RNA pellet was resuspended in 20 μL diethylpyrocarbonate-treated water by passing the solution up and down several times through a pipette tip and then incubated in a water bath or heat block at 55 °C for 10 min. The sample was stored at −80 °C.

### Library preparation and sequencing

Two PCR primers, F515 (5′-GTGCCAGCMGCCGCGG-TAA-3′) and R806 (5′-GGACTACHVGGGTWTCTAAT-3′), were used to target the V4 domain of bacterial 16S rRNA. PCR amplification was performed in a 50-mL reaction volume containing 25 mL 2× Phusion Flash Master Mix (Thermo Fisher, Waltham, MA, USA), 0.5 mM of each forward and reverse primer, and 50 ng DNA template. The reaction conditions consisted of an initial 98 °C for 30 s, followed by 30 cycles of 98 °C for 10 s, 54 °C for 30 s, 72 °C for 30 s, and final extension at 72 °C for 5 min. Amplified products were evaluated by 2% agarose gel electrophoresis and ethidium bromide staining. Amplicons were purified using the AMPure XP PCR Purification Kit (Agencourt, Beckman Coulter, Brea, CA, USA) and quantified using a Qubit dsDNA HS Assay Kit (Thermo Fisher) on a Qubit 2.0 Fluorometer (Thermo Fisher) according to the manufacturer’s instructions. For V4 library preparation, Illumina adapters were attached to the amplicons using the TruSeq DNA Sample Preparation v2 Kit (Illumina, San Diego, CA, USA). Purified libraries were applied for cluster generation and sequencing on the MiSeq system.

Total RNA (150 ng) was used for RNA-seq library construction with the Bacteria ScriptSeq complete kit (Epicentre, Madison, WI, USA). Briefly, ribosomal RNA was removed from total RNA. Next, cDNA synthesis, 5′ tagging, 3′ tagging, and index PCR were sequentially conducted to construct the index library for the Illumina sequencing platform. Libraries were qualified and quantified by Qubit and qPCR. After concentration adjustment, the libraries were mixed and denatured for sequencing.

### Sequence preprocessing

**Fig. 1:**
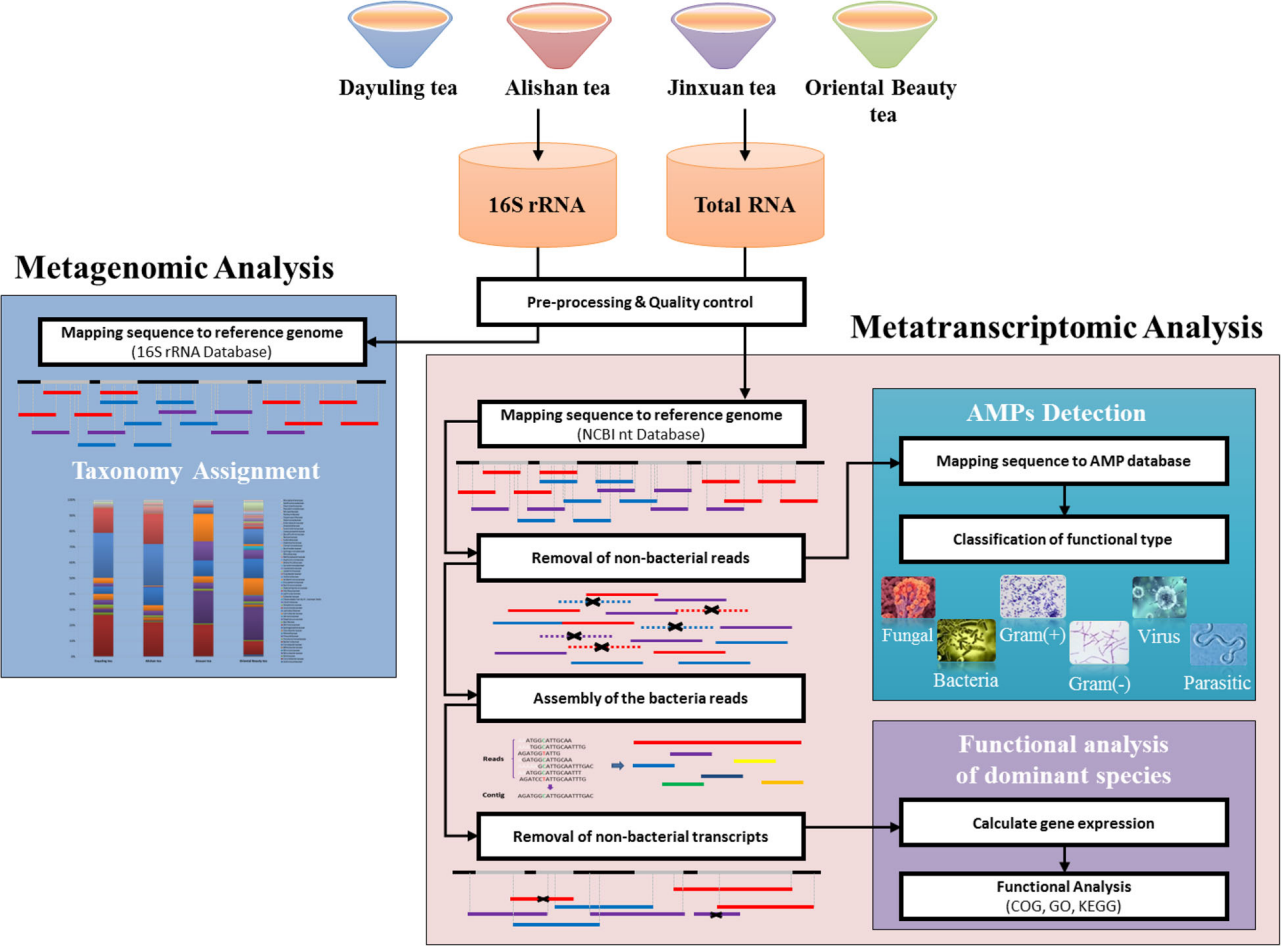
Analytical flowchart of the integrated metagenomic and metatranscriptomic pipeline

Figure 1 shows our analysis flow. Raw reads were preprocessed using the FASTX-Toolkit (a FASTQ/A short-reads pre-processing tools) [24] to trim poor-quality bases. Nucleotides with Phred quality scores lower than 30 were trimmed from the end of the read, and reads longer than 70 nucleotide bases were retained for subsequent filtering. Reads with 70% of their bases, showing quality score higher than 30, were reserved for further analysis. A quality score (Q) less than 30 corresponds to an error probability (P) of 0.001 according to the formula:

$$ \mathrm{Q}=-10{\mathit{\log}}_{10}P $$

### Taxonomic assignment of 16S rRNA sequences

Paired-end sequences were obtained by Illumina sequencing in FASTQ format and the FASTX-Toolkit was applied for sequence quality assessment. Bowtie2 [25] was used to map the paired-end reads to bacterial 16S ribosomal RNA (rRNA) sequences obtained from the NCBI 16S ribosomal RNA sequence database and NCBI nucleotide collection database. The reads were mapped to specific bacteria if sequence similarity exceeded 97% and paired-end reads were aligned to the same reference sequence.

### Functional analysis of transcripts

Next, processed reads from each tea sample were aligned to reference genome sequences using Bowtie2 [21] to bacterial sequences. Reference genome sequences were built from the nt database, which is available from the National Center for Biotechnology Information (NCBI) website, including NCBI genome sequences, Ensembl genome sequences, etc. Because of the high degrees of similarity among bacterial genome sequences, the extracted bacterial reads were assembled into contigs by Trinity [26] and aligned to the reference genome again to discard non-bacterial transcripts. The remaining bacterial transcripts were subjected to taxonomy analysis to identify the distribution of bacteria in each sample. Dominant *E. coli* species were selected for further analysis. To obtain an overview of the functional classes among all samples, we performed Clusters of Orthologous Groups (COG) analysis using BLASTX to map the sequences against the COG database [27]. Sequencing reads were identified by Bowtie2 and BLASTX as being associated with a certain transcript. Those showing the highest identity with the sequences in the COG database were selected to represent each transcript. Additionally, the dominant bacterial species in oolong teas were selected for functional analysis. Gene expression levels were calculated and normalized using RSEM (RNA-Seq by Expectation-Maximization) [28]. Next, gene ontology (GO) analysis was conducted to examine the differences in biological processes, cellular components, and molecular functions of the dominant species among the four tested tea samples. Finally, genes expressed across all four tea samples were selected for KEGG analysis [29].

### Identification of antimicrobial peptides using high-throughput transcriptome data

In this study, we identified 4744 experimentally verified AMPs (Table 1) in published databases, including ADAM [30], CAMP [31], and APD [32]. All collected amino acid sequences of AMPs were transformed into DNA sequences to implement an efficient pipeline for discovering AMPs on NGS reads using the Bowtie2 program. The raw reads of metatranscriptomics data (total RNA) were subjected to quality control and adjustment. After quality control and removing ribosomal RNA and the reads from plants, all reads were mapped to the AMP database and showed a sequence identity of 100%. We provide all of parameters used by those programs in Additional file 1.

**Table 1:** Data statistics of validated AMPs in different functional types

Functional type	Number of AMPs
Antibacterial	3273
Anti-Gram (+)	2684
Anti-Gram (−)	2482
Antifungal	1563
Antiviral	286
Antiparasitic	111
Total	4744

## Results and discussion

### Bacteria taxonomy assignment using 16S rRNA sequences

**Fig. 2:**
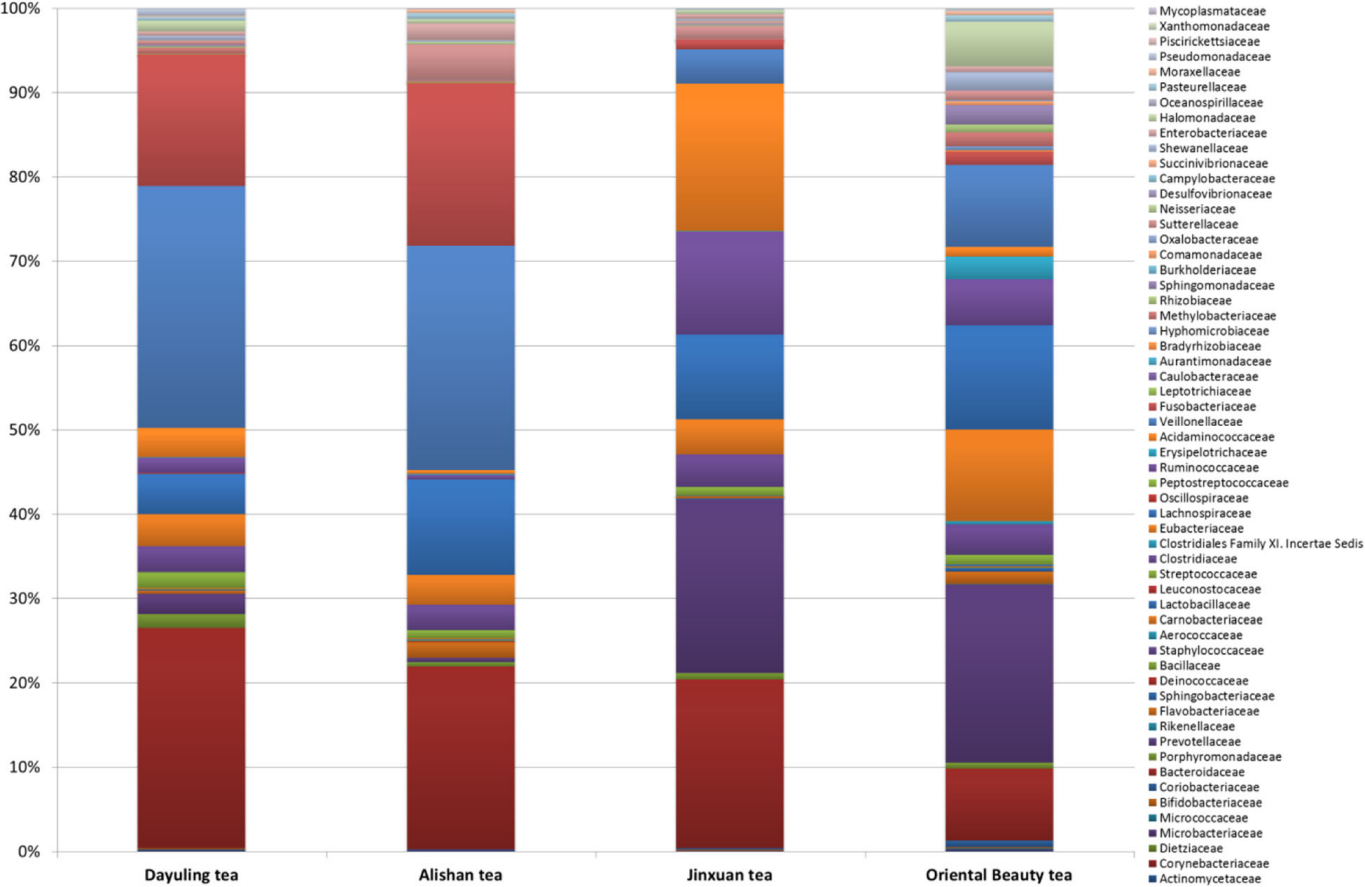
Bacterial communities in four tea samples using 16S metagenomic data

A total of 60,260 sequence reads in the 16S rRNA V4 region were identified using our taxonomic mapping flow from 4 samples with a median read length of 125 base pairs and mean of 15,065 reads per tea sample. Fig. 2 and Table 2 show the bacterial taxonomy assignments at the family level, and operational taxonomic unit tables mapped at different taxonomy levels are provided as Additional file 2. As shown in Fig. 2, bacteria communities present in Dayuling tea and Alishan tea were similar. *Veillonellaceae* belongs to the phylum Firmicutes as the dominant bacteria. The most distinct feature of the *Veillonellaceae* family is that it contains bacteria with a gram-negative cell wall structure within a phylum of gram-positive bacteria, and thus, molecular-based methods may be required to identify the species [33]. Interestingly, the family isolates displayed various resistance patterns to antimicrobial agents [34]. *Bacteroidaceae* was a subdominant family classified in the phylum Bacteroidetes. As previously reported, both *Veillonellaceae* and *Bacteroidaceae* were not affected by tea polyphenols [35]. Polyphenols are natural plant compounds present in green and black tea and are associated with beneficial effects such as the prevention of cardiovascular diseases [36] and several food-borne pathogenic bacteria [37]. However, Oriental Beauty tea exhibited significantly higher bacterial diversity than other teas, with *Prevotellaceae* as the dominant family. De Filippo et al. found *Prevotella* accounted for more than half of the gut bacteria in African children but was absent in European children; this genus enables the host to maximize energy intake from fibers and confers protection against inflammations and noninfectious colonic diseases [38]. Furthermore, metagenomic analysis results showed that the most common bacterial families across all tea types were *Bacteroidaceae* (21.7%), *Veillonellaceae* (22%), and *Fusobacteriaceae* (12.3%). Additionally, the family *Lachnospiraceae* was present in all samples. All species in this family are associated with obesity and may protect against colon cancer by producing butyric acid [39].

**Table 2:** Abundance (number of reads) of bacterial 16S rRNA at the family level for all tea samples

Family	Dayuling tea		Alishan tea		Jinxuan tea		Oriental Beauty tea	
	Reads	%	Reads	%	Reads	%	Reads	%
Actinomycetaceae	35	0.10%	13	0.10%	4	0.00%	7	0.10%
Corynebacteriaceae	7	0.00%	1	0.00%	0	0.00%	1	0.00%
Dietziaceae	1	0.00%	0	0.00%	1	0.00%	0	0.00%
Microbacteriaceae	18	0.10%	8	0.10%	9	0.10%	15	0.20%
Micrococcaceae	19	0.10%	1	0.00%	4	0.00%	6	0.10%
Bifidobacteriaceae	17	0.10%	1	0.00%	12	0.10%	5	0.10%
Coriobacteriaceae	12	0.00%	9	0.10%	18	0.20%	73	0.80%
Bacteroidaceae	7783	26.20%	2841	21.70%	1660	19.90%	771	8.50%
Porphyromonadaceae	474	1.60%	60	0.50%	70	0.80%	63	0.70%
Prevotellaceae	704	2.40%	70	0.50%	1715	20.50%	1918	21.10%
Rikenellaceae	2	0.00%	3	0.00%	5	0.10%	6	0.10%
Flavobacteriaceae	116	0.40%	255	1.90%	15	0.20%	135	1.50%
Sphingobacteriaceae	31	0.10%	5	0.00%	8	0.10%	40	0.40%
Deinococcaceae	0	0.00%	14	0.10%	0	0.00%	0	0.00%
Bacillaceae	20	0.10%	7	0.10%	2	0.00%	6	0.10%
Staphylococcaceae	1	0.00%	0	0.00%	2	0.00%	0	0.00%
Aerococcaceae	0	0.00%	10	0.10%	0	0.00%	0	0.00%
Carnobacteriaceae	31	0.10%	19	0.10%	3	0.00%	11	0.10%
Lactobacillaceae	7	0.00%	1	0.00%	2	0.00%	17	0.20%
Leuconostocaceae	7	0.00%	0	0.00%	0	0.00%	0	0.00%
Streptococcaceae	557	1.90%	137	1.00%	86	1.00%	106	1.20%
Clostridiaceae	917	3.10%	387	3.00%	326	3.90%	330	3.60%
Clostridiales Family XI. Incertae Sedis	13	0.00%	0	0.00%	2	0.00%	36	0.40%
Eubacteriaceae	1115	3.80%	453	3.50%	339	4.10%	986	10.80%
Lachnospiraceae	1416	4.80%	1488	11.40%	834	10.00%	1125	12.40%
Oscillospiraceae	17	0.10%	0	0.00%	2	0.00%	2	0.00%
Peptostreptococcaceae	2	0.00%	0	0.00%	1	0.00%	4	0.00%
Ruminococcaceae	546	1.80%	62	0.50%	1006	12.10%	503	5.50%
Erysipelotrichaceae	24	0.10%	12	0.10%	5	0.10%	233	2.60%
Acidaminococcaceae	1003	3.40%	64	0.50%	1451	17.40%	106	1.20%
Veillonellaceae	8553	28.80%	3487	26.60%	338	4.00%	878	9.70%
Fusobacteriaceae	4621	15.60%	2528	19.30%	89	1.10%	150	1.60%
Leptotrichiaceae	23	0.10%	18	0.10%	1	0.00%	3	0.00%
Caulobacteraceae	0	0.00%	3	0.00%	0	0.00%	0	0.00%
Aurantimonadaceae	3	0.00%	0	0.00%	0	0.00%	0	0.00%
Bradyrhizobiaceae	5	0.00%	0	0.00%	0	0.00%	17	0.20%
Hyphomicrobiaceae	9	0.00%	3	0.00%	3	0.00%	38	0.40%
Methylobacteriaceae	203	0.70%	20	0.20%	16	0.20%	155	1.70%
Rhizobiaceae	58	0.20%	3	0.00%	4	0.00%	86	0.90%
Sphingomonadaceae	64	0.20%	18	0.10%	3	0.00%	210	2.30%
Burkholderiaceae	1	0.00%	0	0.00%	0	0.00%	1	0.00%
Comamonadaceae	14	0.00%	6	0.00%	0	0.00%	45	0.50%
Oxalobacteraceae	9	0.00%	0	0.00%	1	0.00%	9	0.10%
Sutterellaceae	114	0.40%	547	4.20%	129	1.50%	100	1.10%
Neisseriaceae	23	0.10%	37	0.30%	7	0.10%	4	0.00%
Desulfovibrionaceae	31	0.10%	2	0.00%	21	0.30%	11	0.10%
Campylobacteraceae	4	0.00%	0	0.00%	1	0.00%	2	0.00%
Succinivibrionaceae	1	0.00%	0	0.00%	19	0.20%	0	0.00%
Shewanellaceae	122	0.40%	20	0.20%	16	0.20%	194	2.10%
Enterobacteriaceae	142	0.50%	258	2.00%	53	0.60%	62	0.70%
Halomonadaceae	384	1.30%	68	0.50%	40	0.50%	481	5.30%
Oceanospirillaceae	0	0.00%	1	0.00%	0	0.00%	0	0.00%
Pasteurellaceae	142	0.50%	91	0.70%	9	0.10%	69	0.80%
Moraxellaceae	15	0.10%	59	0.50%	3	0.00%	48	0.50%
Pseudomonadaceae	267	0.90%	8	0.10%	11	0.10%	17	0.20%
Piscirickettsiaceae	7	0.00%	0	0.00%	0	0.00%	0	0.00%
Xanthomonadaceae	3	0.00%	3	0.00%	0	0.00%	13	0.10%
Mycoplasmataceae	0	0.00%	2	0.00%	0	0.00%	0	0.00%

### Analysis of transcripts mapped to taxonomy terms

**Fig. 3:**
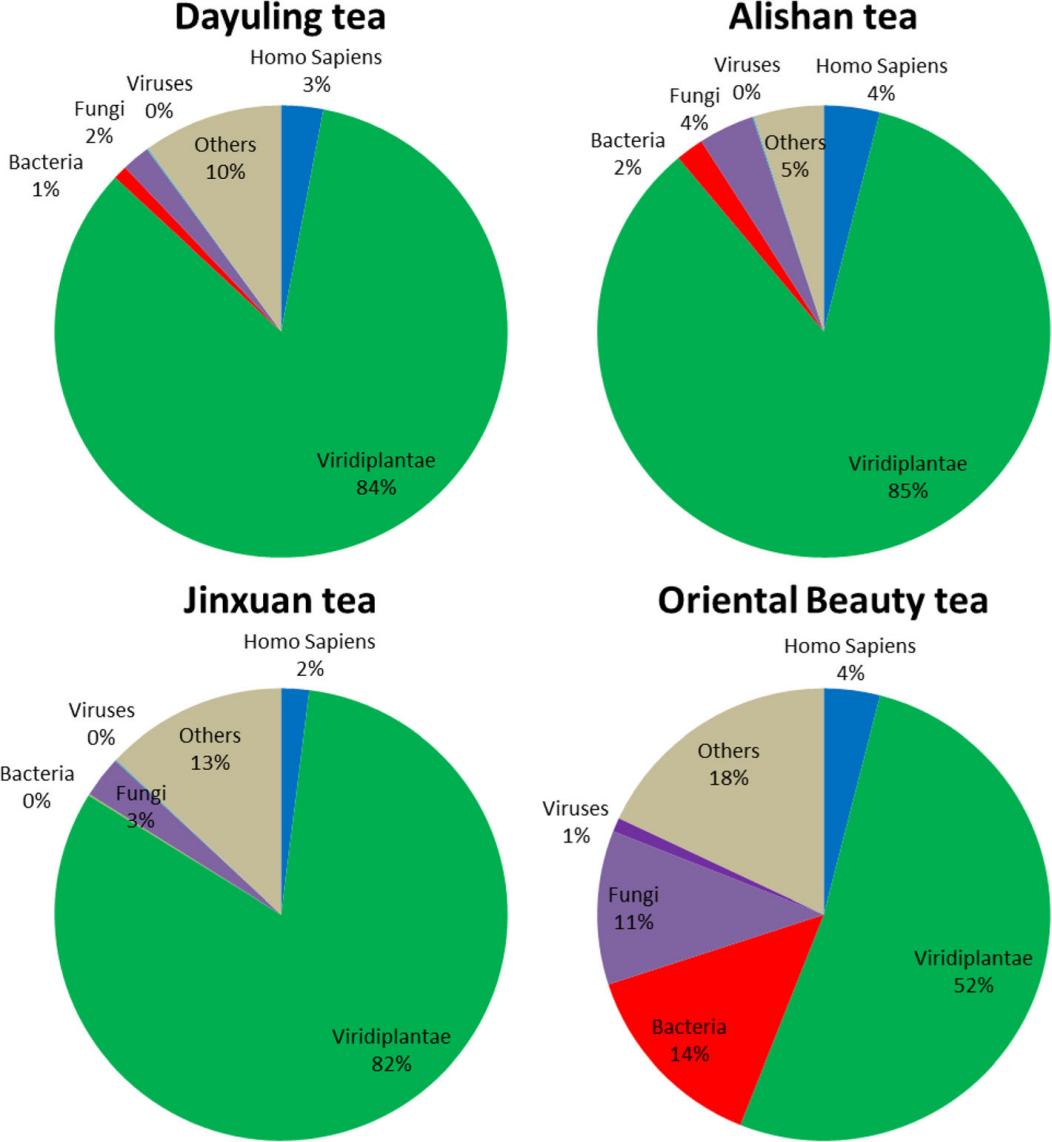
Kingdom assignments of four tea samples using total transcripts

A total of 166,429,720 reads were generated during sequencing, and 80,945,719 reads remained after quality control with a minimum quality cutoff of 20 (Table 3). Reference genome sequence alignments were performed and approximately 80% of the processed reads were assigned a specific kingdom. The read distribution among *Homo sapiens* (3.26%), Viridiplantae (75.41%), bacteria (4.48%), fungi (5.05%), viruses (0.41%), and others (11.40%) in four samples are depicted in Fig. 3. The percentage distribution of reads in different kingdoms among samples was analyzed. The number of reads mapped to *H. sapiens* was more balanced across all samples than those assigned to other groups. For example, as expected, most reads were assigned to Viridiplantae with more than 80% from most samples except for Oriental Beauty tea, in which only half of the processed reads were assigned to Viridiplantae (51.6%). Furthermore, compared to the other tea samples, the differences in the percentage distribution of the reads across different kingdoms was greater for Oriental Beauty tea. The balance distribution of reads belonging to *H. sapiens* across the four tea types can be explained by the short period of contamination from tea farmers during harvest, and the greater biological diversity in Oriental Beauty tea may be related its growth on flat land rather than on the mountains like the other three teas. Among the four samples, 2,038,548 reads were assigned to bacteria and further analyzed.

**Table 3:** Kingdom taxonomic analysis of metatranscriptomic data

	Raw reads	QC reads	QC%	*Homo sapiens*	Viridiplantae	Bacteria	Fungi	Viruses	Others
Dayuling tea	41,894,931	18,100,972	43.21	458,281	11,761,339	125,475	318,656	12,841	1,406,717
Alishan tea	41,152,352	27,498,256	66.82	1,004,044	18,979,598	425,288	831,025	23,668	1,077,615
Jinxuan tea	42,728,182	21,780,331	50.97	276,372	13,713,125	94,797	550,722	3855	2,167,244
Oriental Beauty tea	40,654,255	13,566,160	33.37	349,091	4,940,454	1,392,988	1,047,815	134,282	1,711,734

**Fig. 4:**
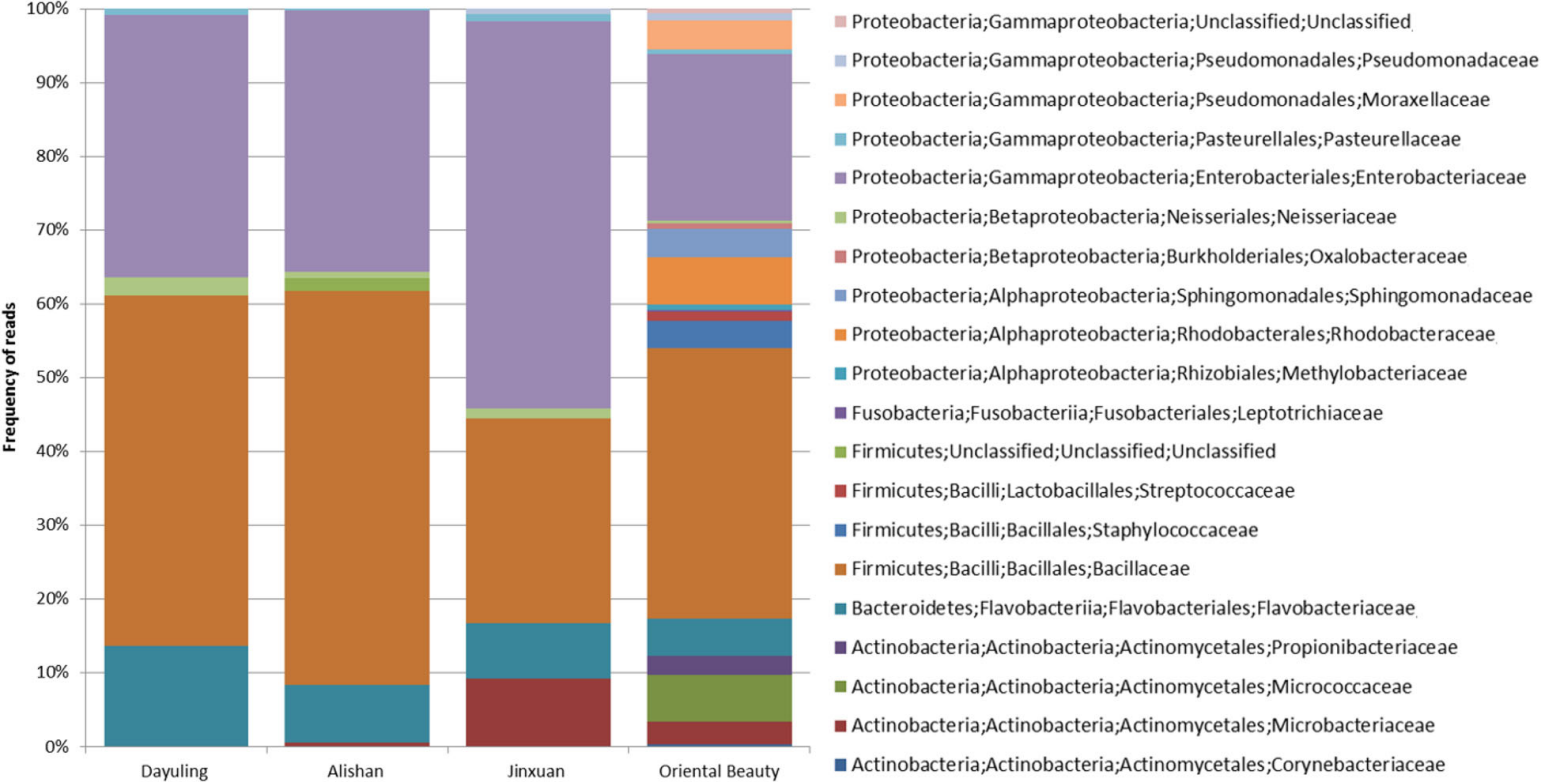
Taxonomic distribution of all bacterial transcripts at family level based on metatranscriptomics analysis

To examine the distribution of bacteria in the four oolong teas, the extracted bacterial reads were assembled using Trinity and aligned to the nt database again to overcome the problem of high sequence similarity among different bacterial genomes. After assembly, 800 contigs were generated and 70 were removed. We counted the reads on each contig to generate the distribution of bacteria in reads as a unit. The top 20 major categories were selected to draw the distribution of bacteria as shown in Fig. 4. The results of taxonomy assignment at the family level indicated that members of *Bacillaceae* and *Enterobacteriaceae* were the most abundant microorganisms, comprising 42% and 36% of the bacterial communities among the tea samples, respectively. Dayuling tea, Alishan tea, and Jinxuan tea shared similarities in the distribution of *Bacillaceae* and *Enterobacteriaceae*, with the former showing approximately 50% and the latter showing 35%. While more than 52% of the bacterial community in Jinxuan tea was composed of *Enterobacteriaceae*, the same family accounted for only 22% of the bacteria found in Oriental Beauty tea. In addition, Oriental Beauty tea showed greater microbial diversity at the family level. For example, 7% of the reads were assigned to *Rhodobacteraceae*, *Micrococcaceae* in the Oriental Beauty tea sample, but were not detected in the other teas. Furthermore, nearly 10% of the reads belonged to *Flavobacteriaceae* in most of the tested tea samples, but the same family was found in less than 5% in Oriental Beauty tea. According to Table 3, the differences in the percentage distribution of the reads across the different kingdoms appeared to be more dramatic for Oriental Beauty tea compared to the other three tea samples. Additionally, by extending our observations to bacterial community analysis using metagenomics and metatranscriptomic data, Shannon’s diversity index [40] was calculated to determine the number of different species in a community while taking into account how evenly the basic entities were distributed among those types, according to the formula:

$$ {\mathrm{H}}^{\prime }=-\sum \limits_{i=1}^R{p}_i\mathit{\ln}{p}_i $$

As provided in Table 4, the index values indicated that Oriental beauty had the highest species diversity compared to the other three samples. This may be because Oriental Beauty tea is grown at lower altitudes than the other three teas, and its leaves have been bitten by the leaf hoppers.

**Table 4:** Shannon’s diversity index of bacterial communities in four oolong teas

Data type	Dayuling	Alishan	Jinxuan	Oriental beauty
Metagenomic Data	2.92	2.73	3.34	3.79
Metatranscriptomic Data	1.76	1.19	1.66	3.08

### Functional analysis of transcripts of dominant bacterial species

**Fig. 5:**
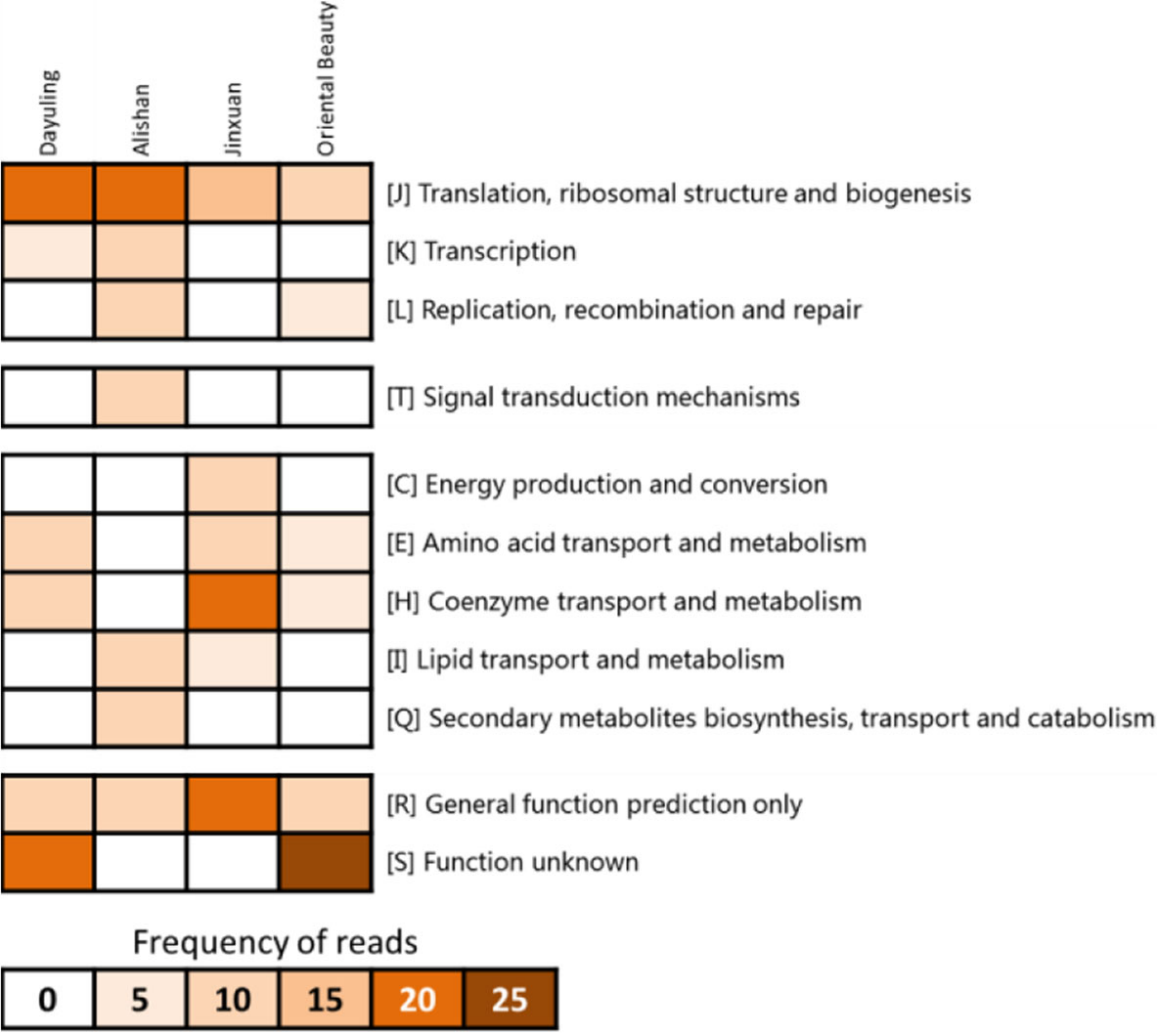
Distribution of COG functional annotations of all bacterial transcripts

To identify the dominant bacterial species for functional analysis, taxonomy was determined at the species level (Additional file 3: Figure S1). Although the microbial diversity of Oriental Beauty tea was greater at the family level, at the species level all four tea samples appeared to share two dominant bacterial species: *E. coli* and *Bacillus subtilis*. To acquire an overview of the functional categories among the tea samples, we assigned each transcript to its corresponding COG category using BlastX (Fig. 5). Most reads were associated with translation, ribosomal structure, and biogenesis; this may be because of conservation of the rRNA sequences among bacterial species. The functions of more than 20% of the reads in Dayuling and Oriental Beauty teas remain unknown. Some of the identified bacterial protein sequences have not been curated in the current COG database, such as a protein in species *Bacillus atrophaeus* present in both Dayuling and Oriental Beauty teas.

**Fig. 6:**
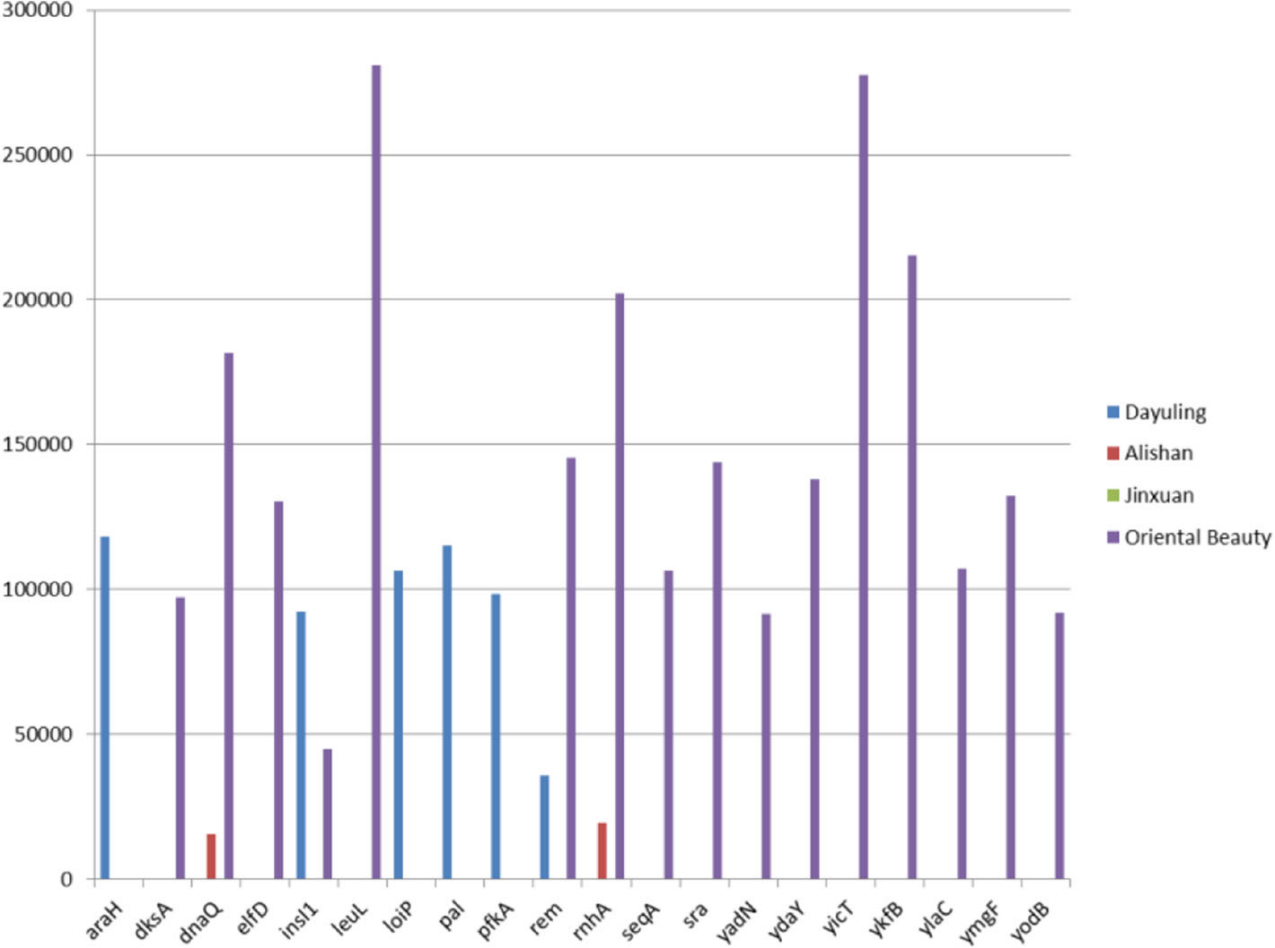
Intensity of top 20 highly-expressed genes in the dominant bacterial species

**Fig. 7:**
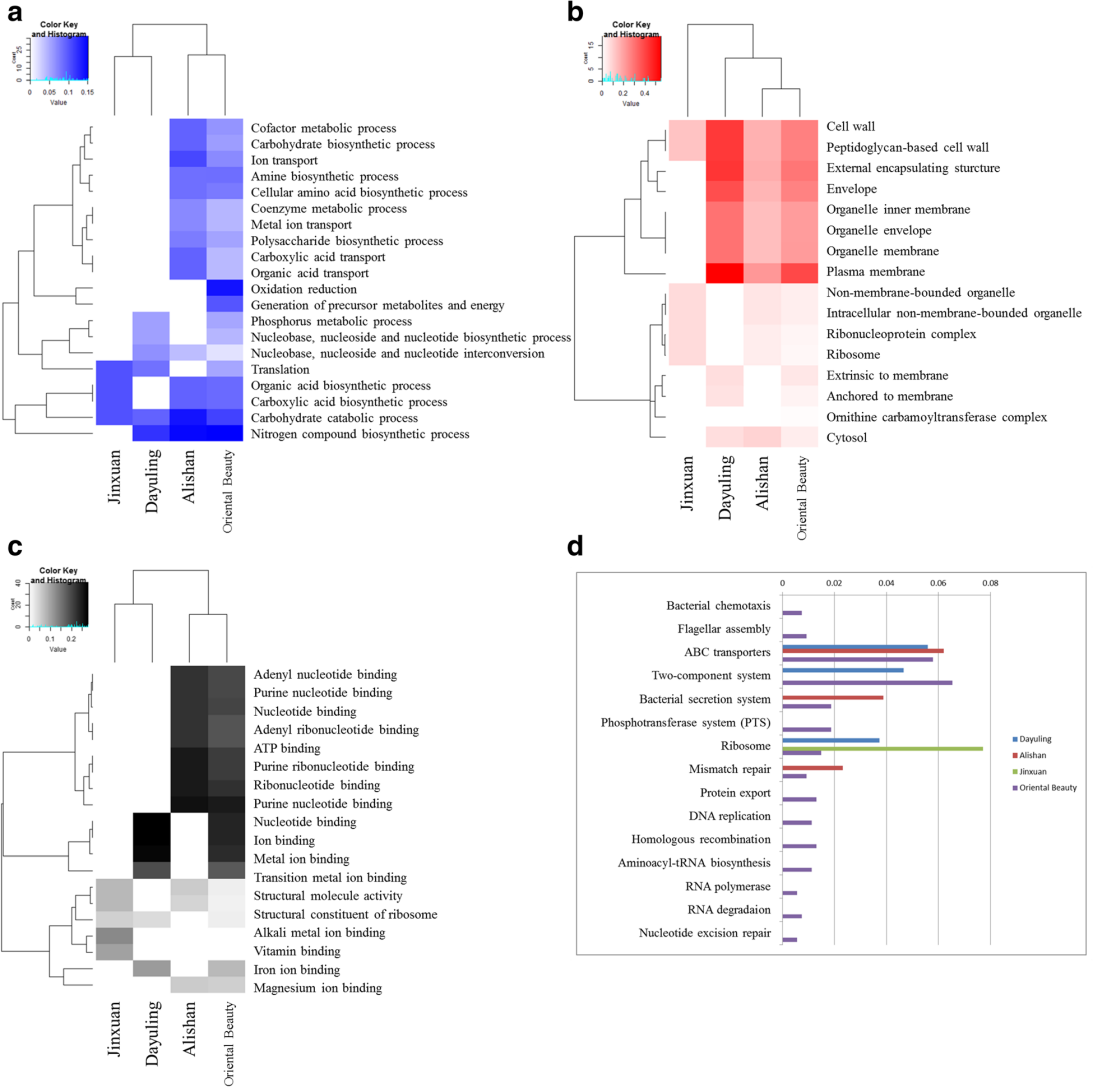
GO analysis and KEGG analysis of transcripts of the dominant bacterial species: **a** Biological process, **b** Cellular component, **c** Molecular function, and **d** KEGG pathway

Due to the abundance of gene annotations in public domain, *E. coli* was subjected to further functional analysis. Based on RSEM gene expression calculations, 962 coding genes were expressed in *E. coli* (Additional file 4). The most highly expressed genes (top 20) are shown in Fig. 6. Gene Ontology enrichment was using DAVID functional annotation tool [41], which performs a gene- annotation enrichment analysis of the set of differentially expressed genes (adjusted fold change > = 2 and FDR < 0.001). MeV is an open source Java application which contains many popular analytical algorithms for clustering and visualization [42]. It has been used to visualize the clustering result of GO among 4 tea samples. This was performed to identify the biological processes, cellular components, and molecular functions associated with these genes (Additional file 5). Figure 7a indicates that while the most actively expressed *E. coli* genes in Dayuling and Jinxuan resembled each other in biological processes, those present in Alishan and Oriental Beauty seemed to be more similar. However, the results of cellular components (Fig. 7b) and molecular functions (Fig. 7c) analysis showed greater differences among the four teas. The KEGG results (Fig. 7d) showed that *E. coli* in Oriental Beauty tea is involved in the largest number of pathways; this may be because of Oriental Beauty tea’s growing environment or heavy fermentation process required to produce the tea. Moreover, *E. coli* in Alishan and Oriental Beauty teas was involved in similar pathways, such as ABC transporters, bacterial secretion system, mismatch repair, purine metabolism, thiamine metabolism, porphyrin and chlorophyll metabolism, as well as alanine, aspartate and glutamate metabolism, nitrogen metabolism, and tryptophan metabolism.

### Identification of natural antimicrobial peptides from bacteria in Taiwanese oolong teas

Many methods and tools can be used to perform metagenomic and metatranscriptomic data analysis. Each approach has advantages and disadvantages, particularly with regard to optimization for different study objectives, such as taxonomic profiling, assessing microbial composition, or identifying functional genes and pathways. For instance, the UPARSE pipeline constructs a set of operational taxonomic unit representative sequences from NGS amplicon reads that can be used to understand the microbial community structure [43]. QIIME is a software that allows users to input raw sequencing data generated on multiple platforms and interprets the data from fungal, viral, bacterial, and archaeal communities and provides a visualized version of the results [44]. MG-RAST server is a SEED-based environment that allows users to upload raw sequence data for automatic analysis of microbial community structure and function [45]. In contrast, we designed a database-assisted system for identifying AMPs and their functional types based on metatranscriptomic analysis of high-throughput transcriptome data. This is a first study to identify natural antimicrobial peptides in bacteria through metagenomic and metatranscriptomic analyses.

**Fig. 8:**
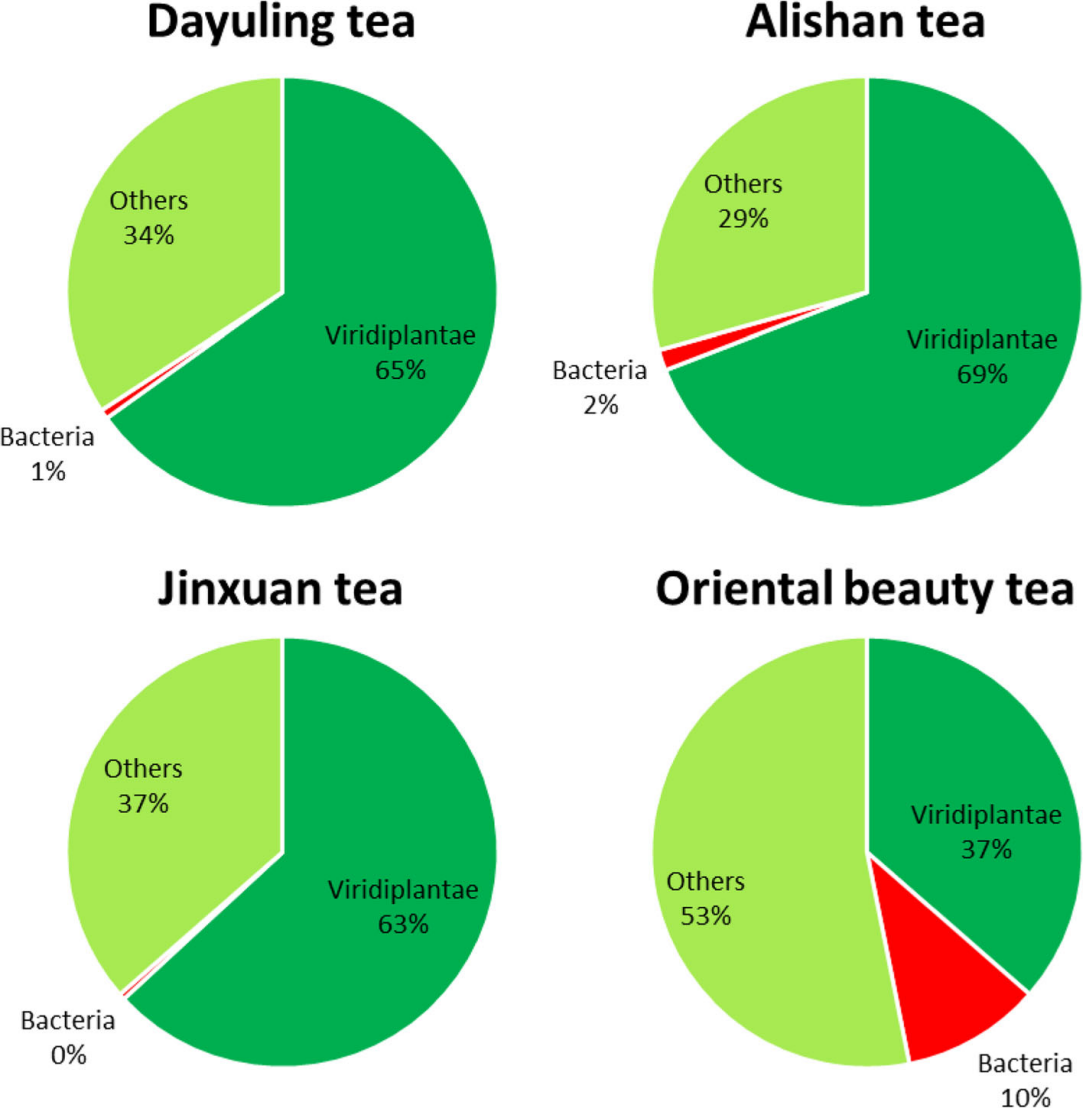
Data distribution of total RNA reads mapped to AMPs in four Taiwanese oolong teas

**Fig. 9:**
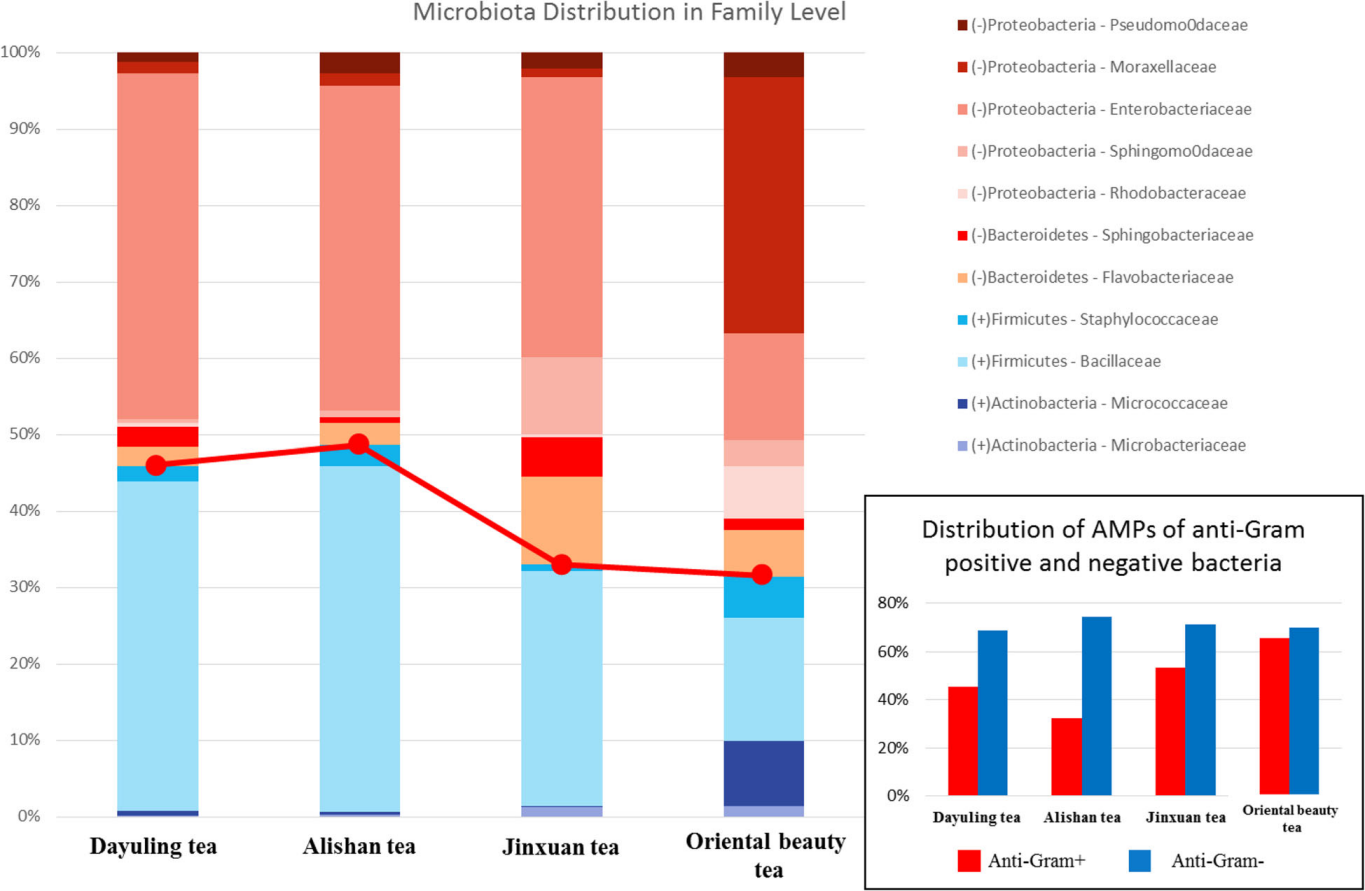
The distribution of anti-gram-positive and anti-gram-negative AMPs is highly correlated with the distribution of gram-positive and gram-negative bacterial in four oolong tea samples

After quality control and removal of non-bacterial reads, the remaining reads were aligned to the AMP dataset, as presented in Tables 5, and 8194 (6.5%), 26,220 (6.1%), 5703 (5.8%), and 10,6183 (7.8%) reads were mapped to AMPs with a sequence identity of 100%. The Oriental Beauty tea sample showed greater microbial diversity at the family level. For example, 7% of the reads were assigned to *Rhodobacteraceae*, *Micrococcaceae* in the Oriental Beauty tea sample, whereas none were found in the other teas. Furthermore, nearly 10% of the reads belonged to *Flavobacteriaceae* in most of the tested tea samples, but the same family was found in less than 5% in Oriental Beauty tea. Oriental Beauty tea contains different bacterial communities possibly because it is grown without pesticides, causing the tea green leafhopper (*J. formosana*) to feed on the leaves, stems, and buds [23]. Figure 8 shows that the Oriental Beauty tea had a higher percentage of bacterial AMPs (10%) when comparing with other three teas. With a more detailed investigation into the functional types of AMPs, it is very interesting that the distribution of anti-gram-positive and anti-gram-negative AMPs is highly correlated with the distribution of gram-positive and gram-negative bacterial in the four oolong tea samples (Fig. 9). Further, dominant bacterial taxa secrete anti-gram positive or negative AMPs against other bacterial species. For instance, a certain percentage of the reads mapped to AMPs belonging to the dominant bacterial family *Moraxellaceae* in Oriental Beauty tea, regardless of the functional types of AMPs that were mapped (Table 6).

**Table 5:** Data statistics of total RNA reads for AMPs mapping

	Dayuling	Alishan	Jinxuan	Oriental beauty
Number of mapped reads (%)	8194 (6.5%)	26,220 (6.1%)	5703 (5.8%)	106,183 (7.8%)
Number of AMPs	876	1130	761	1678

**Table 6:** Number of RNA reads for mapping different functional types of AMP in Oriental Beauty tea

Family	Reads mapped to Anti-Gram (+)	Reads mapped to Anti-Gram (−)
Moraxellaceae	420	399
Pseudomonadaceae	409	179
Sphingomonadaceae	377	358
Enterobacteriaceae	369	251
Bacillaceae	234	81
Sphingobacteriaceae	166	160
Oxalobacteraceae	164	157
Burkholderiaceae	151	109
Methylobacteriaceae	150	142
Caulobacteraceae	149	20
Micrococcaceae	117	109
Propionibacteriaceae	117	73

## Conclusions

In this study, four types of Oolong teas (Dayuling tea, Alishan tea, Jinxuan tea, and Oriental Beauty tea) were collected for 16S ribosomal DNA and total RNA extraction and sequencing. An integrated analysis flow was constructed to identify AMPs and their functional types based on metagenomic and metatranscriptomic analysis of high-throughput transcriptome data. Metagenomics analysis results revealed that bacterial diversity was higher in Oriental Beauty tea than in the other teas. This may be because Oriental Beauty tea leaves are often infested with *J. formosana*, which may contribute to its uniqueness [19] and cause its flavor to be quite different than the other three teas. The results also showed that Dayuling tea and Alishan tea contained similar bacteria communities, and the most common bacterial families across all tea types were *Bacteroidaceae* (21.7%), *Veillonellaceae* (22%), and *Fusobacteriaceae* (12.3%).

Metatranscriptomics analysis results revealed that the dominant bacterial species across all tea types were *E. coli*, *B. subtilis*, and *Chryseobacterium sp. StRB126*. *Escherichia coli* is the most common bacteria and among the most important bacteria in the human gut [46]. Under normal conditions, *E. coli* are not only harmless, but also may be helpful to humans. In addition to facilitating vitamin synthesis and immune system development in humans, they also help prevent invasion by harmful bacteria. *Bacillus subtilis* is a commensal bacterium in the human gut. Previous studies showed that *B. subtilis* produces subtilisin, polymyxin, nystatin, gramicidin, and other active substances during cell growth and that these substances provide significant protection against food-borne pathogens [47]. In addition to the dominant bacteria described above, *Bacillus amyloliquefaciens*, which was found to be present in only Oriental Beauty tea, is known to produce various secondary metabolites including aminoglycosides, β-lactams, polyketides, and small polypeptides, all of which have been shown to inhibit different pathogens [48]. GO analysis and metabolic network analysis was performed to determine the relationship between dominant functional microbial species and the environment.

Additionally, the results indicated that anti-gram-positive AMPs in Oriental Beauty tea had a higher volume of distribution than in the other three teas. Interestingly, we also found that Oriental Beauty tea contained the lowest proportion of gram-positive bacteria at the family level. This may be because Oriental Beauty tea is grown at lower altitudes compared to the other teas. Alternatively, Oriental Beauty tea leaves are often infested with *J. formosana*, which may contribute to its uniqueness.

This is the first study to analyze microbial diversity in Taiwanese oolong teas using metagenomic and metatranscriptomic approaches and to identify natural antimicrobial peptides from bacteria in Taiwanese oolong teas. These results contribute to the current understanding of microbes and their potential functions in oolong tea.

## Additional files

Additional file 1: All of parameters used for analytic programs in each step. (DOCX 13 kb)

Additional file 2: The list of operational taxonomic unit at different taxonomy levels. (XLSX 20 kb)

Additional file 3: Taxonomic distribution of all bacterial transcripts at species level based on metatranscriptomics analysis. (DOCX 268 kb)

Additional file 4: Expression of coding genes in *E. coli* calculating by RSEM. (XLSX 43 kb)

Additional file 5: The results of Gene Ontology enrichment analysis. (XLSX 83 kb)
