# Supplementary material for: Identification of natural antimicrobial peptides from bacteria through metagenomic and metatranscriptomic analysis of high-throughput transcriptome data of Taiwanese oolong teas
Source: BMC Syst Biol. 2017 Dec 21;11(Suppl 7):131. doi: 10.1186/s12918-017-0503-4 (PMC5763296; doi:10.1186/s12918-017-0503-4)
Supplement: Supplementary file 1 — All of parameters used for analytic programs in each step. (DOCX 13 kb) [file 12918_2017_503_MOESM1_ESM.docx]

**Additional file 1 - All of parameters used for analytic programs in each step.**

**1. Quality Control:**

fastq_quality_trimmer -Q 33 -t 30 -i [INFILE] –o [OUTFILE]

fastq_quality_filter -Q 33 -q 30 -p 70 -i [INFILE] -o [OUTFILE]

**2. Bowtie2 align to NT DB:**

bowtie2 –q -N 0 --no-hd --no-mixed --no-unal -x [NT_DB] -U [INFILE] -S [OUTFILE]

**3. Bowtie2 align to silva SSU DB:**

bowtie2 -q -N 0 --no-hd -end-to-end --no-mixed --no-unal -x [SSU_DB] -U [INFILE] -S [OUTFILE]

**4. Blast to AMP DB:**

blastx -query [INFILE] -db [AMP_DB] -evalue 10 -matrix BLOSUM62 -outfmt 6 -out [OUTFILE]

**5. Trinity :**

--method edgeR -P 0.001 -C 2, FDR 0.001, fold change >=2
