# Supplementary material for: Identification of natural antimicrobial peptides from bacteria through metagenomic and metatranscriptomic analysis of high-throughput transcriptome data of Taiwanese oolong teas
Source: BMC Syst Biol. 2017 Dec 21;11(Suppl 7):131. doi: 10.1186/s12918-017-0503-4 (PMC5763296; doi:10.1186/s12918-017-0503-4)
Supplement: Supplementary file 3 — Taxonomic distribution of all bacterial transcripts at species level based on metatranscriptomics analysis. (DOCX 268 kb) [file 12918_2017_503_MOESM3_ESM.docx]

**
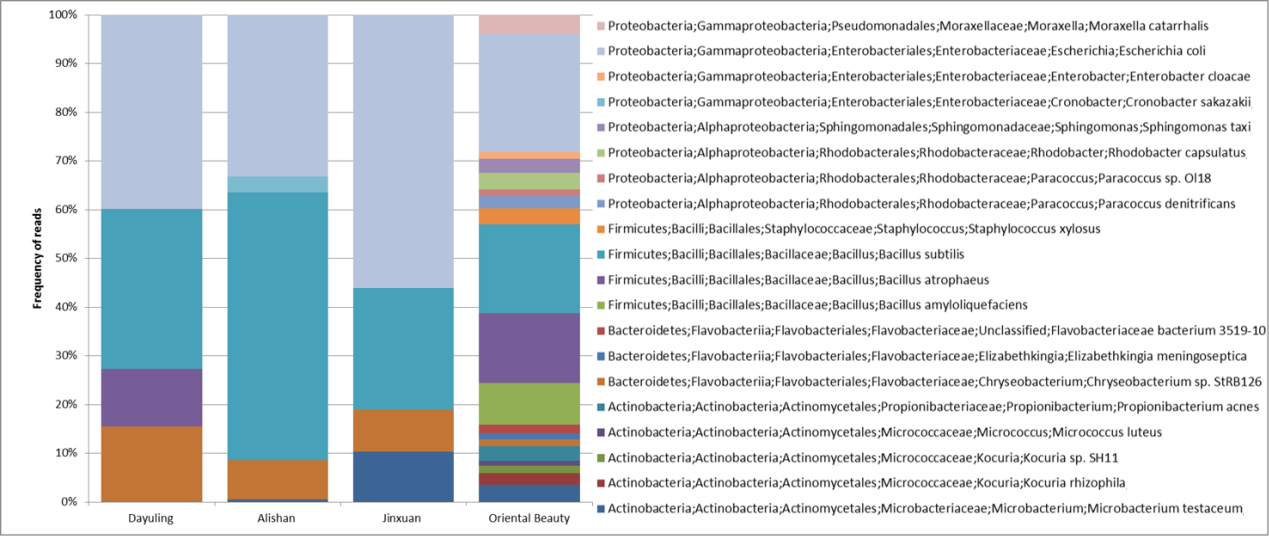
**

**Figure S1. Taxonomic distribution of all bacterial transcripts at species level based on metatranscriptomics analysis.**
